# Potential Protective Effect of Vitamin C on Qunalphos-Induced Cardiac Toxicity: Histological and Tissue Biomarker Assay

**DOI:** 10.3390/biomedicines10010039

**Published:** 2021-12-24

**Authors:** Ayed A. Shati, Mohamed Samir A. Zaki, Youssef A. Alqahtani, Mohamed A. Haidara, Mubarak Al-Shraim, Amal F. Dawood, Refaat A. Eid

**Affiliations:** 1 Department of Child Health, College of Medicine, King Khalid University, Abha P.O. Box 62529, Saudi Arabia; ashati@kku.edu.sa (A.A.S.); Yal-qahtani@kku.edu.sa (Y.A.A.); 2 Department of Anatomy, College of Medicine, King Khalid University, Abha P.O. Box 62529, Saudi Arabia; mszaki@kku.edu.sa; 3 Department of Histology and Cell Biology, College of Medicine, Zagazig University, Zagazig P.O. Box 31527, Egypt; 4 Department of Physiology, Kasr al-Aini Faculty of Medicine, Cairo University, Cairo P.O. Box 11519, Egypt; haidaram@cu.edu.eg; 5 Department of Pathology, College of Medicine, King Khalid University, Abha P.O. Box 62529, Saudi Arabia; malshraim1@kku.edu.sa; 6 Department of Basic Medical Sciences, College of Medicine, Princess Nourah bint Abdulrahman University, P.O. Box 84428, Riyadh 11671, Saudi Arabia; Afdawood@pnu.edu.sa

**Keywords:** quinalphos, vitamin C, albino rats, cardiac toxicity, light and electron microscopy, oxidative parameters, statistical analysis

## Abstract

Insecticides and toxicants abound in nature, posing a health risk to humans. Concurrent exposure to many environmental contaminants has been demonstrated to harm myocardial performance and reduce cardiac oxidative stress. The purpose of this research was to study the protective effect of vitamin C (Vit C) on quinalphos (QP)-induced cardiac tissue damage in rats. Eighteen albino male rats were randomly categorised into three groups (*n* = 6). Control, QP group: rats received distilled water. QP insecticide treatment: an oral administration of QP incorporated in drinking water. QP + Vit C group: rats received QP and Vit C. All the experiments were conducted for ten days. Decline of cardiac antioxidant biomarkers catalase (CAT) and reduced glutathione (GPx) along with increased proinflammatory markers tumour necrosis factor-alpha (TNF-α) and interleukin 6 (IL-6) indicated oxidative and inflammatory damage to the heart following administration of QP when compared to control rats. The light microscopic and ultrastructure appearance of QP-treated cardiomyocytes exhibited cardiac damage. Administration of Vit C showed decreased oxidative and inflammatory biomarkers, confirmed with histological and electron microscopic examination. In conclusion, Vit C protected the heart from QP-induced cardiac damage due to decreased inflammation and oxidative stress.

## 1. Introduction

Pesticides have been shown to increase the concentration of reactive oxygen species (ROS), generating oxidative stress as well as a change in the cell’s prooxidant/antioxidant balance [1]. Pesticide toxicity has been discovered in several research studies and creates oxidative stress in humans and other animals by increasing production of free radicals [2,3,4].

QP is an organophosphate pesticide frequently used in agriculture and the cattle industry. Approximately 0.1 per cent of pesticide reaches its intended target, with the rest dissipating in the environment. QP and its metabolites can survive for long periods in water, soil, or plants, posing serious harm to exposed animals and humans [5]. QP inhibits acetylcholinesterase activity, resulting in acetylcholine build up at synaptic and neuromuscular junctions [6]. According to recent research, poisoning with QP and its intermediate metabolites promotes oxidative stress generated by free radical formation [7]. As a consequence, pollution created by the indiscriminate use of QP is a major source of concern.

Everywhere around the world, cardiac dysfunction is a primary source of infection and mortality. The development of cardiovascular problems is increasingly related to environmental contamination [8,9].

Carotenoids and vitamins C, A, and E are the most frequent natural antioxidants derived from food [10]. As a result, the significance of natural antioxidants in limiting oxidative injury as a component in the pathology, physiology, and histology of a wide range of disorders has recently piqued interest [11,12]. The antioxidants vitamins C and E are accessible as dietary supplements and are essential components in nearly all biological functions. Vit C is a chain-breaking antioxidant that is soluble in water [13]. This, however, has caused it to become one of the most frequently utilised and cheapest non-enzymatic antioxidant chemicals for the prevention of oxidative damage [14]. Both physiological ROS and reactive nitrogen species are easily scavenged by it [15]. The best sources of Vit C include vegetables, fruits, and organ meats (including kidney and liver), while muscle meats and most seeds are insufficient [16].

In humans and animals, Vit C has been demonstrated to decrease the haematological and biochemical alterations caused by organophosphate insecticides [17,18,19]. This widely available, inexpensive, non-toxic antioxidant is particularly effective in reducing the deleterious effects of most xenobiotics [20].

Organophosphorus has been shown to induce cardiac damage by causing significant changes in the form of disorganisation of the cardiomyocytes with increased interstitial spaces between these fibres. It also causes increased masses of collagen fibres among the cardiomyocytes and around the congested blood vessels associated with extensive collagen fibre deposition [21].

The aim of this research is to evaluate the potential protective role of Vit C in QP-induced cardiac toxicity in rats.

## 2. Materials and Methods

### 2.1. Ethics Statement

The use of animals in research was governed by the standards set forth by King Khalid University’s College of Medicine in Saudi Arabia. All experimental procedures were approved by the medical research ethical committee at King Khalid University, Abha, KSA on Jan 8 2021 (ECM#2021-6008). The National Institutes of Health’s international criteria for the care and use of laboratory animals are followed in these guidelines (NIH Publications No. 8023, revised 1978).

### 2.2. Experimental Design

Eighteen mature male Sprague–Dawley rats weighing 200–250 g were divided into three groups of six animals each. Each group’s treatment plan looked like this:

Group I (Control): rats were housed in a rat cage and given distilled water by oral gavage every day at 8 a.m. for ten days.

Group II (QP insecticide treatment): rats received a forceful oral dose of 14 mg kg^−1^ QP [o,o-diethyl-o-(2-quinolinyl phosphorothioate)] in 100 μL distilled water, which was purchased as “Sequin” 25 per cent EC from the source (Sudarshan Chemical Industries Ltd., Pune, Maharashtra 411001, India). For ten days, QP was given via oral gavage at 8:00 a.m. each day.

Group III (QP insecticide and Vit C, co-administered): rats received oral administration of Vit C supplied as pure crystals (Sigma Chemical Company, St. Louis, MO, USA) from E. Merck Science, a branch of EM industries Inc., Darmstadt, West Germany, four hours after QP administration (14 mg kg^−1^ in 100 μL distilled water) for ten days at a dose of 20 mg/kg/day (i.e., twice the human approved therapeutic dose of 10 mg/kg BW/day).

All of the animals were sacrificed between 10:00 and 12:00 a.m. one day after the last day of treatment to avoid any diurnal changes in hormone and neurotransmitter concentrations.

### 2.3. Specimen’s Processing and Staining for Light Microscopy (LM)

The hearts were dissected quickly and preserved for 24 h in 10% neutral buffered formalin. Before being dehydrated with alcohol at increasing concentrations, the right atrial tissues were washed in a 0.1 M phosphate buffer solution. Paraffin was used to embed the specimens. Myocardial sections (5 μm thick) were deparaffinised for 15 min with two changes of xylene and rehydrated with descending alcohol grades (100 per cent, 90 per cent, and 70 per cent). The sections were stained with haematoxylin and eosin (H & E) (Abcam, Boston, MA, USA) to look at the overall morphology [22].

### 2.4. Tissue Preparation for Transmission Electron Microscopy (TEM)

The cardiac specimens were extracted and inspected under an electron microscope. Tiny pieces were treated with 2.5 per cent glutaraldehyde for 24 h before being rinsed with phosphate buffer (0.1 M, PH 7.4). Post fixation was performed at 4 °C for 1–2 h in 1 per cent osmium tetroxide buffered to PH 7.4 with 0.1 M phosphate buffer, washed in phosphate buffer to remove excess fixative, dehydrated through rising grades of ethanol, and cleared in propylene oxide. To create gelatine capsules, the specimens were immersed in Araldite 502. By heating the capsules to 60 °C, polymerization was achieved. Ultrathin sections (100 nm) were created and picked up on uncoated copper grids using a JEOL ultramicrotome. After double staining with uranyl acetate and lead citrate, sections were examined and photographed using a TEM (JEM-1011, JEOL Co., Tokyo, Japan) operating at 80 kV [22,23].

### 2.5. Parameters Processing and Estimation

Animals were sacrificed by cervical dislocation at the end of the experiment, and the heart was taken to assess various oxidative stress markers.

### 2.6. The Antioxidant Situation of Heart Homogenate

Cardiac samples were homogenised in 100 mM tris–HCl (pH 7.4) and stirred at 12,000 g at 4 °C over 12 h. The supernatant was used to calculate cardiac antioxidant parameters such as GPx, which was determined [24], and CAT activity, which was assessed using the Aebi method [25]. Cardiac GPx and CAT were measured by colorimetric methods. Spectrophotometric measurement of MDA (indicative of lipid peroxidation) was performed using the thiobarbituric acid substrate assay (TBARS Assay Kit, Item No. 10009055, Cayman Chemical Company, Ann Arbor, MI, USA) as an indicator.

### 2.7. TNF-α and Il-6 Levels in the Blood

After the animals were sacrificed, blood levels of TNF-α (ELISA kit BIOTANG INC., Cat. No. R6365, Lexington, MA, USA) and IL-6 (ELISA Kit, BIOTANG INC., Cat. No. R6365, Lexington, MA, USA) were measured using ELISA kits according to the manufacturer guidelines (ELISA Kit, BIOTANG INC., Cat. No. R6365, Lexington, MA, USA).

### 2.8. Statistical Analysis

The mean standard deviation of means for *n* = 6 experiments is shown. ANOVA was used to analyse data, completed by a post hoc Tukey HSD test with Bonferroni correction for repeated measurements to ascertain statistical significance at a *p* < 0.05 level. Systat for Windows, version 13, was used to conduct the statistical analysis (Systat Inc., Evanston, IL, USA).

## 3. Results

### 3.1. Biochemical and Statistical Analysis

**Figure 1:**
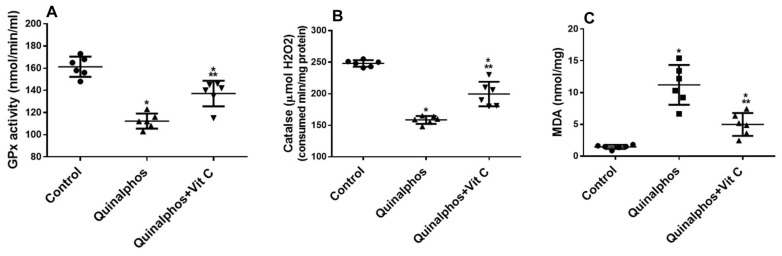
Vit C protects rats from QP-induced oxidative stress and decreases antioxidant biomarkers. At the end of the study, the values of GPx (**A**), CAT (**B**), and MDA (**C**) in heart homogenates were assessed in the three groups of rats included in this study: control, QP, and QP + Vit C. The findings are the mean (±SD) f; *n* = 6. Experiments were carried out in triplicate. * *p* < 0.05 in comparison to control group, ** *p* < 0.05 in contrast to QP group.

**Figure 2:**
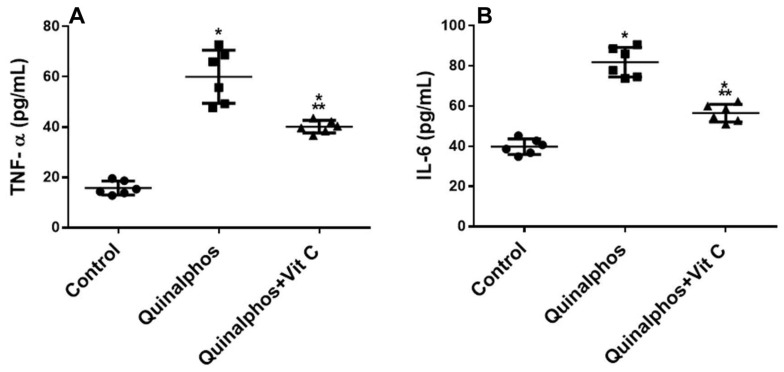
In rats, Vit C suppresses QP-induced inflammatory indicators. The levels of TNF-α (**A**) and IL-6 (**B**) in the blood of three groups of rats were measured at the end of the experiment: control, QP, and QP + Vit C. The findings are the mean (SD) for each group; *n* = 6. Experiments were carried out in triplicate. * *p* < 0.05 in comparison to control, ** *p* < 0.05 in comparison to QP group.

In all rat groups, we looked at antioxidant levels in tissues to assess how much Vit C hindered the regulation of these indicators (Figure 1A,B) and inflammation (Figure 2A,B). QP considerably (*p* < 0.05) reduced the antioxidant biomarker GPx (Figure 1A) and CAT (Figure 1B). Vit C significantly increased both antioxidant enzymes in comparison to the QP group.

As indicated in Figure 2A,B, QP enhanced heart tissue injury considerably (*p* < 0.05). Vit C decreases both inflammatory biomarkers in comparison to the QP group.

### 3.2. Light Microscopy

**Figure 3:**
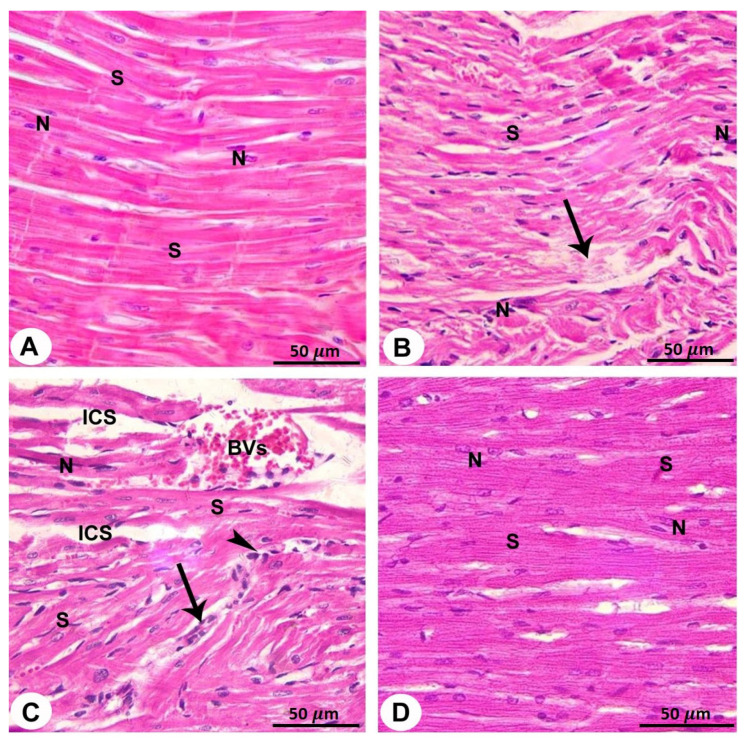
Light micrographs of the rat atrial muscles have been utilised from all groups (X400; H&E). (**A**). Control rat’s group displaying acidophilic sarcoplasm in cylindrical muscle fibres, a large, oval, central vesicular nucleus (N), and branching anastomosing cardiac muscle fibres. (**B**). QP-treated rat’s group exhibiting cardiac muscle fibre breakdown and discontinuance (arrow) and regions of pale acidophilic cytoplasm with pyknotic nuclei. (**C**). QP-treated rat’s group showing widened intracellular spaces (ICS) between cardiac fibres; blood vessels (BVs) are congested, with red blood cell extravasation (arrow) in the interstitium and mononuclear cellular infiltration (arrowheads). (**D**). QP + VIT-C-treated rat’s group exhibiting cardiac muscle fibres with centrally oval vesicular nuclei in a near-normal configuration. Scale bar 50 µm.

Disorganisation and discontinuance of cardiac muscle fibres, enlarged intracellular gaps among cardiac muscle fibres, congestion of blood vessels, and red blood cell extravasation were all observed in sections collected from QP-treated rats. Furthermore, myocardial sections from this group revealed pale, relatively homogeneous acidophilic cytoplasm areas with either no nuclei or highly stained pyknotic nuclei. Moreover, in the heart of QP-treated rats, mononuclear cellular infiltration was visible between muscle fibres (Figure 3B,C and Table 1).

Heart sections taken from rats who received Vit C and QP demonstrated intact muscle fibres with nearly normal oval vesicular central nuclei (Figure 3D and Table 1).

### 3.3. Electron Microscopy

#### 3.3.1. Control Group

**Figure 4:**
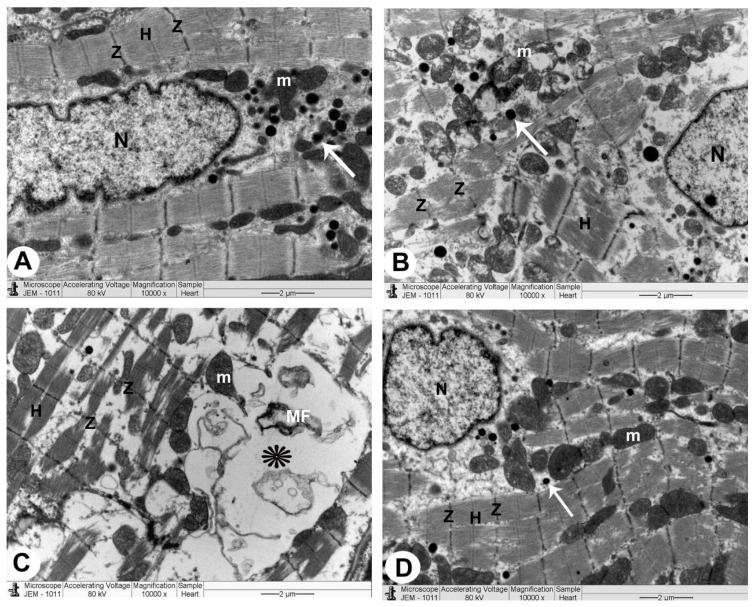
The right atrium is imaged using TEM in all groups of rats (atrionatriuretic factor (ANF) granules): (×10,000). (**A**) The control rat group has a normal myocyte structure with compact cytoplasm packed with the intact striped appearance of myofilaments (Z and H), a nucleus (N), and mitochondria (m). There are also granules of ANF that are tightly compacted (arrows). (**B**,**C**) QP-treated rat’s group exhibiting myofilament cytoplasm damage, muscle band fragmentation (Z and H), and pleomorphic mitochondria (m). There are also a few ANF granules (arrows) and abnormally shaped nuclei (N). In addition, damaged myelin figures (MF) can be detected in the cytoplasm (asterisk). (**D**) QP + Vit C-treated rat’s group revealing myofibrils with a striped appearance with distinct bands (Z and H), nucleus (N), and mitochondria which are all healthy (m). ANF granules (arrows) can also be seen.

**Figure 5:**
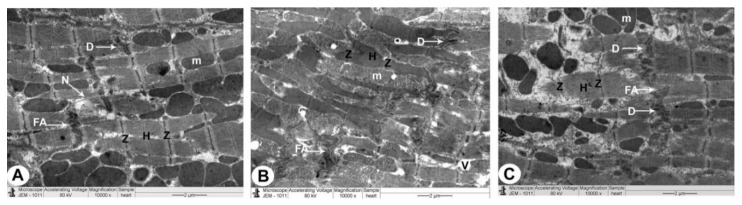
The right atrium is imaged using TEM in all groups of rats (intercalated disc): (×10,000). (**A**) Control rat’s group displaying a healthy intercalated disc that contains fascia adherence (FA), desmosomes (D), and gap junction (nexus) (N). Myocytes have a typical striped appearance with definite bands (Z and H) and mitochondria (m), as well as a compact cytoplasm which is filled with healthier myofibrils. (**B**) QP-treated rat’s group exhibiting intercalated discs that have been destroyed among disturbed myofibril, with the breakdown of muscle bands (Z and H) and pleomorphic mitochondria (m); fascia adherence (FA) and desmosomes (D) are markedly irregular. (**C**) QP + Vit C-treated rat’s group demonstrating a healthy intercalated disc. Fascia adherence (FA) and desmosomes (D) are to be noted. There are also mitochondria (m), along with intact myofibrils with a striped appearance with clearly visible bands (Z and H).

**Figure 6:**
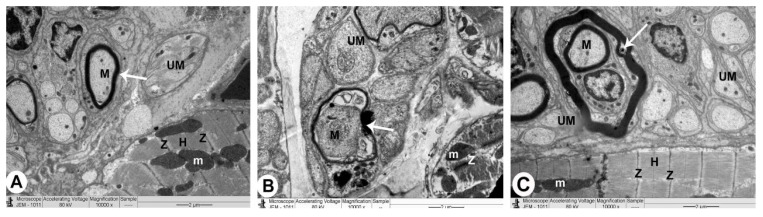
The right atrium is imaged using TEM in all groups of rats (nerve endings): (×10,000). (**A**) Control rat’s group displaying normally myelinated (M) containing intact myelin and unmyelinated (UM) nerve fibres in the interstitial area between intact myofilaments (Z and H) and mitochondria (m) and their striped appearance with obvious bands. (**B**) QP-treated rat’s group exhibiting pleomorphic myelinated (M) nerve fibres with disrupted myelin (arrow) and unmyelinated (UM) nerve fibres in the interstitium among apoptotic myofilaments. (**C**) QP + Vit C-treated group showing preserved myelinated (M) nerve fibres with intact myelin (arrow) and unmyelinated (UM) nerve fibres in the interstitial area.

The sarcoplasm of a normal myocyte was made up of longitudinal arrays of cylindrical myofibrils that split to pass around the nucleus, generating a biconical juxtanuclear region with cell organelles. In this location, mitochondria were plentiful, with cristae packed densely together. Sarcomeres could be seen between two Z lines, separated by the H band, which was a pale area. Intercalated disks had transverse sections with a number of fasciae adherence and desmosomes and longitudinal sections with plenty of gap junctions. In the interstitial space between the myocytes, ANF granules with intact myelin sheath of myelinated nerve fibres and unmyelinated nerve fibres were also seen (Figure 4A, Figure 5A, Figure 6A and Table 1).

#### 3.3.2. QP-Treated Group

The nuclei were irregular in the majority of atrial myocytes, while some were profoundly recessed, with clusters of heterochromatin and a broadening of the juxtanuclear region. Myocytes with broken myofibrils also showed isolated areas of myofibril fragmentation and lysis, as well as sarcoplasmic vacuolisation, particularly in the juxtanuclear region. The mitochondria were arranged in an irregular pattern with a wide range of sizes, and the matrix density was uneven. It was sometimes swollen and had partially destructed cristae. Between the deteriorated myofibrils with muscle band fragmentation (Z and H), damaged intercalated discs with marked irregularity of fascia adherence and desmosomes were detected. In the interstitial area between apoptotic myofibrils, a few ANF granules, pleomorphic myelinated with damaged myelin, and unmyelinated nerve fibres were reported (Figure 4B,C, Figure 5B, Figure 6B, and Table 1).

#### 3.3.3. QP- and Vit-C-Treated Group

The appearance of atrial muscle fibres in all ultrathin sections was substantially identical to that of the QP group. Myofibrils were nearly uniformly disseminated, with an average size and regular striation pattern, and the mitochondrial cristae and thick matrix were mostly intact. Some myocyte nuclei were eccentric, slightly uneven, and had a conspicuous nucleolus, while others were oval euchromatic with an average juxtanuclear area. The clear bands (Z and H) of the intercalated disks, ANF granules, and myelinated and unmyelinated nerve fibres in the interstitial area appeared undamaged (Figure 4B,C, Figure 5B, Figure 6B, and Table 1).

## 4. Discussion

Our results showed that there is a decrease of the antioxidant enzymes GPx and CAT associated with increased MDA in the QP group. MDA is an indicator of lipid peroxidation and is measured as a biomarker of oxidative stress (OxS). This is consistent with the data that showed that OxS is caused by increased production of ROS and is associated with a reduction in antioxidant defences and altered cellular redox status. When there is an imbalance between the creation and scavenging of oxygen-free radicals, OxS develops [26].

Enzymes such as GPx and CAT are part of the antioxidant defence system. GPx is a lipid peroxidation checker and a free radical scavenger. The transformation of reactive oxygen radicals is mediated by the catalytic enzyme CAT. As a result, any change in antioxidant machinery components will result in an accumulation of ROS, causing oxidative insult to cells [7].

Antioxidant enzymes are the cell’s first line of defence against oxidation-induced cellular damage. The mechanism by which QP would enhance oxidative stress and cellular problems may be attributed to decline of CAT and GPx activities in cardiac tissues which will lead to peroxidation of lipid, an accumulation of ROS within the cell, leading to degenerative changes [27].

OxS causing structural and functional alterations in the cellular biomolecules and the cell membrane is the result of the development of complications through elevated levels of inflammatory biomarkers [28] This is consistent with our results; where there is an increase of the proinflammatory cytokines in QP hearts compared to control hearts, TNF-α and IL-6 were found to be increased in the serum of the QP group. According to our findings, TNF-α and IL-6 may have a significant effect in causing myocardial damage. Data showed that TNF infusion causes myocardial damage, myocyte shrinkage, and left ventricular hypertrophy in rats [29]. TNF-α has previously been found to be produced in large quantities by cardiac myocytes [30]. As a result, TNF-α produced by cardiac myocytes is expected to play a crucial role in heart tissue malfunction. However, Takada et al. clarified that TNF-α and IL-6 mRNA can also be generated by macrophages identified in the heart and serum of rats [31].

The light microscopic appearance of QP-treated cardiomyocytes exhibited fragmented cytoplasm with missing striations in the current study. According to some researchers, the myofibrillar injury may occur as a result of mitochondrial dysfunction; this disruption of myofibrillar structure and composition may be attributed to differences in calcium absorption and adenosine triphosphate production [32].

The risk begins with an overabundance of free radicals in the cell; furthermore, mitochondrial ROS may function as a modulatable redox indicator, altering the performance of a range of tasks in the mitochondria, cytosol, and nucleus in a reversible manner [33]. As a result, LM images revealed black, apoptotic nuclei following QP treatment, but TEM images displayed irregular, deeply indented nuclei with clumps of heterochromatin. These changes indicate that the cells are less active or possibly unhealthy [34,35].

Our results showed that mitochondria were structured randomly with different shapes in the QP-treated rats, and the matrix composition was heterogeneous. It was sometimes swollen and had partially destructed cristae. Mitochondria appear to be an important redox signalling node due to the outflow of ROS superoxide radicals. In addition, the mitochondrial matrix is in charge of oxidative phosphorylation, iron sulphur centres, and haem synthesis [36]. The mitochondrial oxidative effect can be attributed to outer membrane permeability, allowing intermembrane gap proteins like cytochrome C to leak into the cytosol and trigger the cell’s apoptotic mechanisms [37].

Our results showed that QP-treated groups have abundant intercalated discs (ICDs) and substantial irregularity in fascia and desmosomes [38]. A cell adhesion protein (α-E-catenin) is hypothesised to be implicated in the binding of the cadherin-based adhesion complex to the actin filaments and is found at the adherent junction [39].

We also showed that in the QP group, there were pleomorphic myelinated nerve cells with damaged myelin and unmyelinated nerve cells. Endoneurial edema is generated by the blood-nerve barrier and the capillary endothelial cell layer, which increases capillary permeability and local pressure gradients, potentially resulting in cellular hypoxia [40].

Atrial myocytes from rats revealed ANF granules, which were few and mostly destroyed in the QP-treated group. ANF, a diuretic and natriuretic substance found in rat atrial granules, was discovered to be a substantial component [38,41]. ANF granules were effectively managed as a universal hormonal system that interacts with other neuropeptides and endocrines created by other cell components to enhance the accuracy of documentation and “memory” in the heart [41,42,43].

Cells generate endogenous enzymatic and/or non-enzymatic antioxidant defences to protect themselves from free radical damage. Even though cells have an effective defence system, they may become overloaded under stressful situations. Antioxidants help to maintain redox equilibrium by reducing the production of ROS, scavenging them, and interfering with the alterations they trigger [44].

Vit C protects the body by acting as a necessary cofactor for numerous enzymes and as an effective, if not the most effective, antioxidant. Vit C combines directly with superoxide and maintains redox equilibrium; it is no longer just a scavenger, but rather a sensor and effector of redox-regulated pathways in the cell [45]. Vit C has been shown to reduce the load of free radicals by scavenging oxygen, reducing hydroperoxide, and stabilising free radicals into neutral and harmless molecules [43]. Vitamin C’s antioxidant and metabolic properties may aid to lower the risk of cardiovascular diseases [46].

The protective effect of Vit C against ROS-induced inflammation in tissues is consistent with our results which showed that administration of Vit C caused an increase in antioxidant enzymes (GPx and CAT) and decreased MDA, which is a biomarker of increased lipid peroxidation and oxidative stress. This was confirmed with histological examination where Vit C administration in QP + Vit C-treated group showed near-normal configuration of cardiac myocytes.

TEM examination also confirms these results; administration of Vit C to rats that received QP showed healthy intercalated discs and normal mitochondria along with the striped appearance of intact myofibrils with clearly visible Z and H bands.

Many chemicals are thought to be developmental neurotoxicants, although only a small percentage has been evaluated. Widespread exposure of the human population to chemicals contributes to many neurodevelopmental disorders [47].

A common route by which different agents produce similar neurotoxic outcomes is through the production of ROS [48,49].

Organophosphate pesticide (chlorpyrifos) was documented to produce OxS, cell loss, altered neurodifferentiation, and adverse effects on brain development.

Vit C was suggested as a preventive agent in Pb neurotoxicity through its antioxidant effect [50]. Consistent with this study, our TEM examination showed administration of QP caused pleomorphic myelinated nerve endings with damaged myelin and unmyelinated nerve cells. Administration of Vit C to the QP group showed preserved undamaged myelinated nerve endings with intact myelin, and unmyelinated nerve cells in the interstitial area, which confirm the neuroprotective effect of Vit C.

From the above, it is clear that QP-induced cardiac damage would cause heart failure as evident from increased oxidative stress and inflammation, which was confirmed with histological and TEM examination. This is consistent with the study which showed that clinical manifestations of cardiac damage in different subjects include heart failure, arrhythmia, cardiogenic shock, and sudden death [51]. Vit C as a preventive measure may be prescribed for those who are exposed to organophosphorus compounds.

In conclusion, it is possible to deduce from the findings of this study that Vit C co-administration in QP-treated rats protects against QP-induced cardiac damage due to its ability to increase antioxidant enzymes and decrease oxidative stress and proinflammatory cytokines.

## Figures and Tables

**Table 1:** Histological and ultrastructural parameters of control; QP-treated rats and QP + Vit-C-treated rats.

No.	Aspects	Control Rats	QP-Treated Rats	QP + Vit-C-Treated Rats
1	Atrionatriuretic factor (ANF) granules	Small electron-dense granules focused mainly in the centre of the cell, between both plasma membrane and peripheral muscle fibres, and close to the nucleus.	Only a minority and most of the granules of various sizes are disturbed.	Normal distribution and normal size granules
2	Intercalated discs	Normal	Damaged	Intact
	• Fascia adherence	Healthy	Discrepancies	Normal
	• Macula adherence	Normal	Abnormalities	Normal
	• Gap junctions (Nexus)	Intact	Lost	Reconstruction
3	Cardiomyocytes	Normal architecture	Degenerated myofibrils	Intact architecture
	• Myofibrils	Preserved sarcomeres	Damaged sarcomeres	Normal sarcomeres
	• Z and H bands	Clear	Disrupted	Intact
	• Mitochondria	Intact	Disorganised	Preserved
4	Nerve endings in interstitial areas	Normal	Damaged	Intact
	• Myelinated fibres	Intact with intact myelin sheaths	Damaged with ruptured myelin sheaths	Normal with intact myelin sheaths
	• Unmyelinated fibres	Intact	Detached	Intact

## Data Availability

The data that support the findings of this study are available upon request from the corresponding author.
